# Cortical Mechanisms of Single-Pulse Transcranial Magnetic Stimulation in Migraine

**DOI:** 10.1007/s13311-020-00879-6

**Published:** 2020-07-06

**Authors:** Joseph O. Lloyd, Kim I. Chisholm, Beatrice Oehle, Martyn G. Jones, Bright N. Okine, Adnan AL-Kaisy, Giorgio Lambru, Stephen B. McMahon, Anna P. Andreou

**Affiliations:** 1 Headache Research-Wolfson CARD, Guy’s Campus, King’s College London, London, UK; 2 Department of Neurorestoration, Wolfson Centre for Age-Related Diseases, King’s College London, London, UK; 3 Zenith Neurotech Ltd, London, UK; 4 Pain Management and Neuromodulation Centre, Guy’s and St Thomas’s NHS Foundation Trust, King’s Health Partners, London, UK; 5 Headache Centre, Guy’s and St Thomas’s NHS Foundation Trust, King’s Health Partners, London, UK

**Keywords:** Migraine, transcranial magnetic stimulation, GABA, glutamate, cortex

## Abstract

Single-pulse transcranial magnetic stimulation (sTMS) of the occipital cortex is an effective migraine treatment. However, its mechanism of action and cortical effects of sTMS in migraine are yet to be elucidated. Using calcium imaging and GCaMP-expressing mice, sTMS did not depolarise neurons and had no effect on vascular tone. Pre-treatment with sTMS, however, significantly affected some characteristics of the cortical spreading depression (CSD) wave, the correlate of migraine aura. sTMS inhibited spontaneous neuronal firing in the visual cortex in a dose-dependent manner and attenuated l-glutamate-evoked firing, but not in the presence of GABA_A/B_ antagonists. In the CSD model, sTMS increased the CSD electrical threshold, but not in the presence of GABA_A/B_ antagonists. We first report here that sTMS at intensities similar to those used in the treatment of migraine, unlike traditional sTMS applied in other neurological fields, does not excite cortical neurons but it reduces spontaneous cortical neuronal activity and suppresses the migraine aura biological substrate, potentially by interacting with GABAergic circuits.

**Electronic supplementary material:**

The online version of this article (10.1007/s13311-020-00879-6) contains supplementary material, which is available to authorized users.

## Introduction

Migraine is a common [1], highly disabling [2], primary headache condition, characterised by debilitating, long-lasting headaches, associated with sensory symptoms [3]. Migraine pathophysiology involves a considerable network of brain neuronal pathways [4]. Although the hypothalamus is now suggested to play a crucial role in attack initiation, the cortex has been also highlighted in a recent fMRI study as an area with an important role in migraine initiation [5]. Electrophysiological studies have shown altered cortical activity in the ictal and pre-ictal phases, particularly within the occipital cortex [6, 7]. Migraine aura is generally considered to be due to a wave of cortical spreading depression (CSD), emanating from the occipital visual cortex [8]. Work in animal models of migraine suggests that CSD may give rise to migraine headache through peripheral or central mechanisms, or both [9–11].

Non-invasive neuromodulation techniques have more recently emerged as alternatives to pharmaceuticals in the management of migraine, helped by the development of patient-friendly devices and favourable tolerability profiles [12, 13]. Single-pulse transcranial magnetic stimulation (sTMS) has been used in clinical neurology for decades. Transcranial magnetic stimulation (TMS) induces weak electrical currents in the underlying cortex through electromagnetic induction [14]. A portable hand-held sTMS device has been approved by the FDA for the acute and preventive treatment of migraine. sTMS was found to be an effective acute treatment in a randomised, double-blind, parallel group, sham-controlled trial in migraine with aura patients, with sustained pain-free response rates at 24 h and 48 h post-treatment [15]. An open-labelled study has also shown its efficacy as an acute treatment in migraine without aura patients [16]. Recent post-market studies suggested that sTMS may be an effective, well-tolerated treatment option for migraine prevention [17, 18].

sTMS has been long used in neurophysiological settings and is believed that when applied over the cortex at a sufficient strength, it preferentially activates interneurons oriented in a plane parallel to the brain surface. This placement over the motor cortex leads to a trans-synaptic activation of pyramidal cells evoking descending volleys in the corticospinal tract, and induction of phosphenes when placed over the visual cortex. Contrary to sTMS, repetitive transcranial magnetic stimulation (rTMS) is believed to change and modulate cortical activity beyond the stimulation period. The physiological basis of rTMS after-effects has not been clearly identified, but the mechanisms may resemble long-term potentiation (LTP) and long-term depression (LTD). Recent studies in healthy subjects and in depression patients suggested that rTMS modulates connectivity strength within the default mode network, as well to the subgenual anterior cingulate cortex and the ventral striatum [19, 20]. How sTMS in migraine interferes with cortical neurons in the visual cortex and with other neuronal networks has not been clearly defined. Interestingly, clinical experience with sTMS-treated migraine patients indicates that these patients do not report the induction of phosphenes while, when the sTMS device is positioned over the motor cortex, it does not elicit any muscle response. A previous study showed that sTMS blocks mechanical and chemically induced CSD in animal models of migraine. sTMS was also shown to attenuate spontaneous and C-fibre-evoked activity of third-order thalamic neurons, potentially through modulation of corticothalamic connections [21]. However, the actions of the sTMS parameters used clinically in migraine treatment on cortical activity have not been previously examined. Hence, it remains unknown if sTMS using these parameters can excite or inhibit the occipital cortex and how it contributes to the inhibition of CSD. In the current study, we aimed to investigate the cortical effects of sTMS using the actual migraine treatment configuration.

## Methods

### Animals and Ethical Approvals

All experiments were performed in accordance with the United Kingdom Home Office Animals (Scientific Procedures) Act (1986). Experiments were designed and performed as per ARRIVE recommendations. Experiments were carried out in adult male Sprague-Dawley rats (*N* = 115, 250–350 g; Charles River, UK), in adult Snap25-2A-GCaMP6s-D mice (*N* = 17 [8 males and 9 females], 25–35 g; Jackson Laboratory, Bar Harbor, USA) and in adult C57BL/6 mice (*N* = 5 [3 males and 2 females], 25–35 g; Jackson Laboratory, UK). Both rats and mice were housed on a 12-h/12-h light/dark cycle with food and water available *ad libitum*. All experiments were performed under general anaesthesia and terminated by anaesthesia overdose and/or cervical dislocation.

### Surgery

#### Mice

Snap25-2A-GCaMP6s-D and C57BL/6 mice were used for *in vivo* imaging experiments. General anaesthesia was induced through the intraperitoneal injection of 0.3 ml urethane (12.5% in saline), and additional doses were titrated based on the depth of anaesthesia which was assessed by hindlimb withdrawal to toe pinch and corneal reflex activity, until surgical depth was achieved. Core body temperature was maintained at 37 °C through a homeothermic mat with an associated rectal probe (Harvard Apparatus). A tracheal catheter was installed to ensure maintenance of a clear airway, and the mice breathed spontaneously. The skull surface was exposed, and the parietal bone on one side was secured to a custom-made head mount stage, for stability and alignment, using orthodontic acrylic resin and cyanoacrylate glue (Lang, Wheeling), leaving the other side accessible. A cranial window was drilled between bregma and lambda to expose the cortex. The window was sealed using a glass coverslip and Vaseline grease.

#### Rats

General anaesthesia was induced with intraperitoneal injection of 60 mg kg^−1^ pentobarbital sodium (Merial, UK). Supplementary anaesthesia was maintained with continuous intravenous infusion of pentobarbital (12–15 mg kg^−1^ h^−1^). A tracheotomy was performed to permit ventilation of the animal, and end-tidal expired CO_2_ was monitored and maintained between 3.5 and 4.5% (Capstar-100, CWE). The left femoral vein and artery were cannulated to allow for constant intravenous infusion of anaesthetic and monitoring of blood pressure, respectively. Adequate anaesthesia was gauged by the absence of toe pinch withdrawal and eye-blink reflexes and gross changes in blood pressure. Core temperature was monitored and maintained near 37 °C using a homoeothermic blanket system (TC-1000, CWE). The animal was fixed on a non-magnetic stereotaxic frame (Kopf Instruments). Craniotomies were performed, and the dura mater was incised to expose the occipital and parietal cortex for electrophysiological recordings/stimulations.

#### *In Vivo* Cortical Imaging

Snap25-2A-GCaMP6s-D or C57BL/6 mice were randomly positioned under an Eclipse Ni-E FN upright confocal/multiphoton microscope (Nikon, UK), at an ambient temperature of 32 °C, and imaged through air, using a Plan Fluor dry 4× (0.13) objective (Nikon) and a GaAsP NDD detectors. Images were acquired at a frame rate of between 0.25 and 2 Hz, depending on experimental requirements and signal strength. To visualise blood vessels, C57BL/6 mice were given injection via the tail vein with 0.05 ml of dextran tetramethylrhodamine (MWt., 70,000; lysine fixable solution, 10 mg ml^−1^; Thermo-Fisher Scientific, UK). To obtain confocal images, a 488-nm argon ion laser was used and a Coherent Chameleon II laser tuned to 920 nm for multiphoton imaging. GCaMP signal was imaged at 500–550 nm and dextran signal at 570–620 nm. Time series recordings were taken with a fully open pinhole for maximal signal collection.

### Spontaneous Neuronal Activity Recordings

Extracellular spontaneous activity from single units in the visual cortex was recorded using a glass-insulated tungsten microelectrode (Kation Scientific, USA) with an impedance of ~ 1 MΩ. Signals were amplified to a gain of 5000 (NeuroLog, Digitimer, UK; NL104, NL106), fed through a Humbug noise eliminator (Quest Scientific, Canada) and bandpass filtered (500–4000 Hz, NL125/NL126). The conditioned signal was displayed on analogue and digital storage oscilloscopes and digitised for storage on a computer using a Micro 1401-3 with Spike2 software (CED, UK). At the end of the experiments, the recording location was lesioned by passing electrical current through the recording electrode. Brains were collected upon termination of the experiment and sliced using a microtome, and sections were examined under a microscope for the presence of a lesioned spot.

### Microiontophoresis

Seven-barrelled carbon fibre electrodes were used to deliver drug solutions and a dye for marking recording sites, using a microiontophoresis current generator (Dagan 6400; Dagan Corporation, USA), while simultaneously recording single-unit neuronal activity. Micropipette barrels were randomly filled with l-glutamate, 200 mM, pH 7.4 (Sigma-Aldrich, UK); (−)-bicuculline methochloride, 100 mM, pH 3.5 (Tocris Cookson, UK); 100 mM 2-hydroxysaclofen (GABA_B_ antagonist; Santa Cruz Biotechnology, Inc., Dallas, US) at pH 9.0; pontamine sky blue (PSB) dye, 2.5% *w*/*v* in 100 mM sodium acetate, pH 6.5 (BDH Laboratory Supplies, UK); and NaCl, 1.0 M, pH 7.5, for automated current balancing. Microiontophoretic barrels had resistances of 20–150 MΩ. l-Glutamate (10–90 nA) and saclofen (60–70 nA) were ejected as an anion and retained with small positive currents; bicuculline (45–90 nA) and PSB were ejected as cations and retained with small negative currents. Neurons were excited by the programmed ejection of l-glutamate in timed pulses. The ejection (10–20 s) and rest (10–20 s) periods were adjusted for each cell to produce a sustainable and reproducible response with firing rates of typically 10–50 spikes s^−1^. In the experiments with bicuculline, the drug was applied at 45–90 nA. Spontaneous and evoked neuronal activity was simultaneously recorded via the carbon fibre recording electrode, and signals were filtered, amplified and processed as above. PSB was ejected at 2 μA at the end of each experiment to mark the recording site. Brains were collected upon termination of the experiment and sliced using a microtome, and sections were examined under a microscope for the presence of a PSB spot. Where a PSB spot could not be identified, the electrode track was traced to identify the recording site.

### Induction and Recordings of Cortical Spreading Depression

A small cranial window was drilled over the frontal cortex and another prepared over the visual cortex. The latter was used to induce CSD as described below. Cortical steady potential was recorded via a 1.5-mm borosilicate glass microelectrode with a tip diameter of 1–2 μm containing 3 M NaCl, inserted to a depth of 50–100 μm in the frontal cortex. The microelectrode was coupled to a reference Ag/AgCl electrode placed in contact with exposed neck muscle. The signal was amplified (NL102G headstage, NL102 DC amplifier) and displayed on a computer. Warm mineral oil was used to prevent exposed areas drying.

CSD was induced electrically, using a constant current isolated stimulator (DS3, Digitimer) with a concentric bipolar stimulating electrode (tip diameter, 25 μm; FHC, USA) inserted to a depth of ~ 100 μm into the visual cortex. Amplitude and duration of the electrical stimulating pulse was increased until a CSD wave was triggered, as previously described [22], in order to determine the induction threshold.

### Single-Pulse Transcranial Magnetic Stimulation

A bespoke *in vivo* single-pulse transcranial magnetic stimulator and coil were developed by eNeura Inc. (USA) for use in scientific research. The stimulator consists of a high-voltage power supply, an energy-storage capacitor bank and an 11-mm-diameter circular coil, enclosed in a 14-mm-diameter insulated cup to minimise transfer of heat from the coil generated during TMS. The coil is connected to the stimulator via a rise-time adjustment box with a 170-μs tap. This mirrors the time rise of the sTMS clinical device for treatment of migraine. The stored energy in the capacitor bank rapidly discharges a high current pulse passing through the coil. This creates an individual, transient, monophasic magnetic pulse of 170-μs rise time and a width of 360 ms. Capacitor recharge takes ~ 5–60 s, depending on the voltage supply. Pulse intensity is variable up to a maximum intensity of ~ 1.1 T, measured using a gauss meter at 5 mm distance from the coil (Table 1). As the strength of the magnetic pulse field falls rapidly at a bigger distance (Table 1), the sTMS coil was held on a Kopf mounted holder and placed over the visual cortex ~ 5 mm from the surface of the skull.

**Table 1:** Magnetic field strength in tesla (T) as measured by a gauss meter at 5 mm, 10 mm and 15 mm distances of the coil, connected to a 170-μs tap-on rise-time adjustment box

Voltage (set point)	5 mm	10 mm	15 mm
50	0.093	0.025	0.011
100	0.193	0.050	0.023
200	0.377	0.113	0.049
300	0.565	0.170	0.070
400	0.744	0.225	0.100
500	0.924	0.282	0.123
600	1.103	0.316	0.146

### Experimental Protocols

#### *In Vivo* Cortical Imaging

**Fig. 1:**
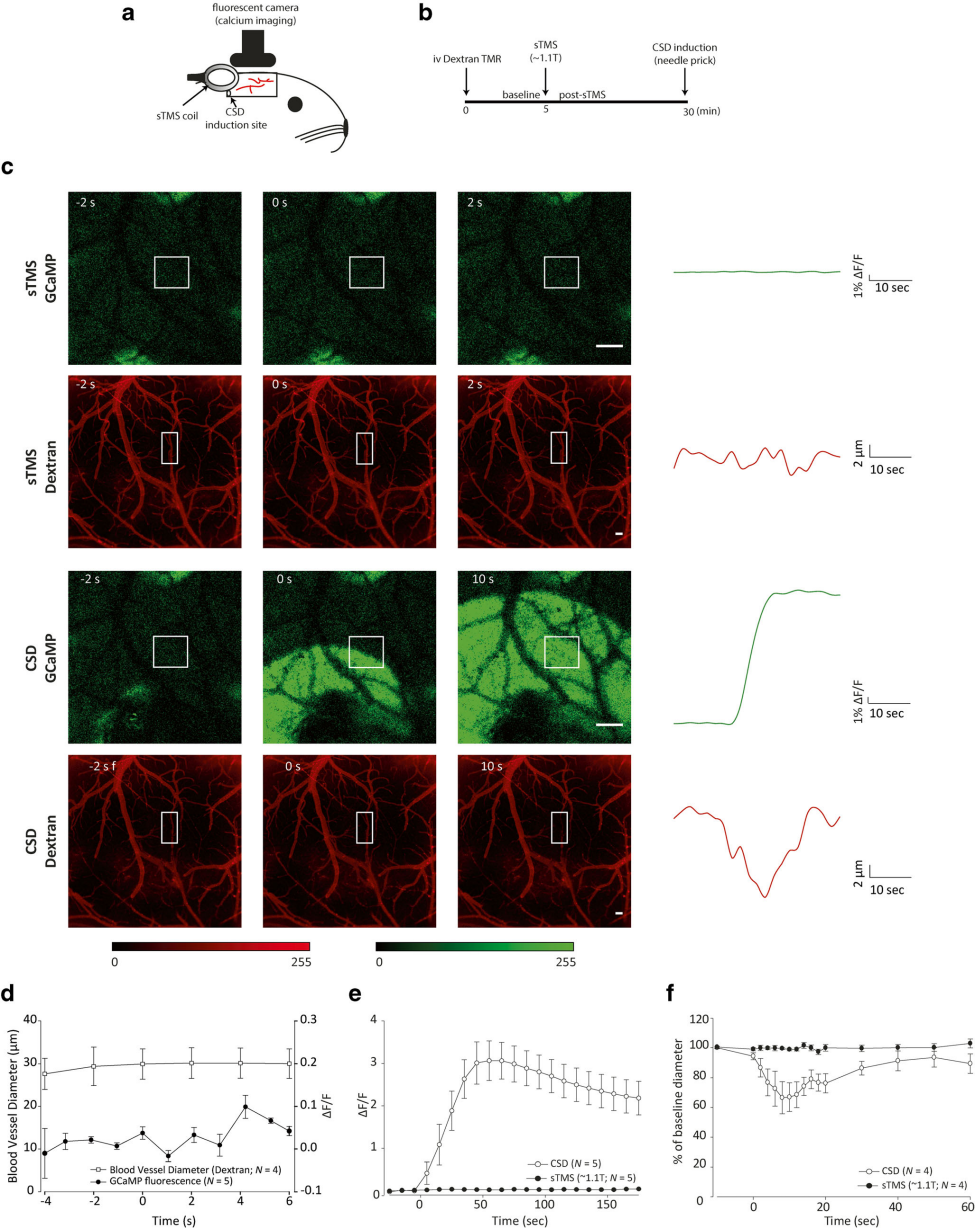
sTMS does not excite cortical neurons. (A) Experimental setup in the Snap25-2A-GCaMP6s-D mouse model. Cortical activity was recorded with *in vivo* calcium imaging through a cortical window from bregma to lambda. The sTMS coil was positioned above the cortex, close to the recording site. CSD was induced by mechanical stimulation (needle prick) at the caudal aspect of the cortical window. (B) Illustration of the experimental protocol employed in C57BL/6 mice injected with dextran, in order to visualise cortical blood vessels. CSD was induced before the end of the experiment as a positive control of cortical blood vessel diameter changes. (C) Examples of cortical imaging frames in a Snap25-2A-GCaMP6s-D (scale bar, 100 μm) or in dextran-injected C57BL/6 mouse (scale bar, 200 μm) immediately before (baseline) and after the cortical event of either a single pulse at 1.1 T sTMS or mechanically induced CSD. Traces show averaged change in fluorescence of a region of interest (indicated by the small squared window). (D) A single sTMS pulse at 1.1 T had no immediate effect on cortical blood vessel diameter or on neuronal firing indicated by lack of change in fluorescence. (E) A mechanically induced CSD evoked a large increase in GCaMP fluorescence, indicating activation of superficial cortical neurons in 2A-GCaMP6s-D mice. Two consecutive sTMS pulses at 1.1 T sTMS had no effect on GCaMP fluorescence over time. Time point 0 indicates when the cortical event (sTMS or CSD) was triggered. (F) A mechanically induced CSD significantly changed cortical blood vessel diameter, but two consecutive sTMS pulses at 1.1 T had no immediate effect on cortical blood vessel diameter. Time point 0 indicates when the cortical event (sTMS or CSD) was triggered

Given the stimulus artefact produced by the sTMS machine and recorded in electrophysiological experiments, it remains unclear whether or not sTMS per se excites cortical neurons at these intensities. To answer this question, *in vivo* calcium imaging in Snap25-2A-GCaMP6s-D mice was employed to visualise the immediate effect of sTMS on cortical calcium uptake as a marker for cortical activity. Baseline recordings of the GCaMP signal were taken from the visual cortex for 5 min before a sTMS pulse (~ 1.1 T) was applied caudal to the cortical window. Image recording was continued. As a positive control of neuronal excitation, CSD was induced via needle prick stimulation of the cortex, by inserting a 30-G needle to a depth of ~ 1 mm into the cortex at the caudal aspect of the cortical window before termination of the experiment (Fig. 1A, B).

To assess if sTMS can induce any changes to cortical vasculature, C57BL/6 mice were injected with dextran and the diameters of two blood vessels per animal were measured. Baseline measurement were recorded for at least 5 min and following two 1.1-T sTMS pulses applied as before. As a positive control of blood vessel diameter changes, a CSD was induced before completion of the experiment by inserting a 30-G needle ~ 1 mm into the cortex through a gap left at the rear of the cortical window for this purpose (Fig. 1A, B).

**Fig. 2:**
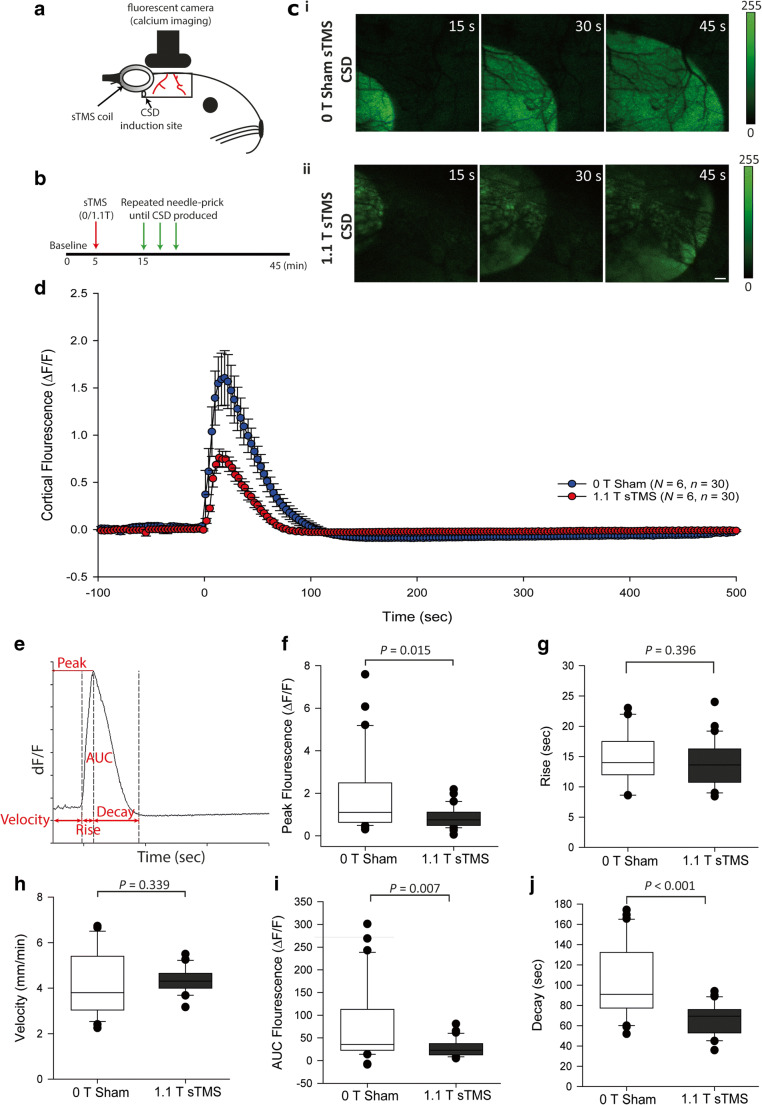
sTMS alters properties of the CSD wave. (A) Experimental setup in the Snap25-2A-GCaMP6s-D mouse model. Cortical activity was recorded with *in vivo* calcium imaging through a cortical window from bregma to lambda. The sTMS coil was positioned above the cortex, close to the recording site. CSD was induced by mechanical stimulation (needle prick) at the caudal aspect of the cortical window. (B) Illustration of the experimental protocol employed in Snap25-2A-GCaMP6s-D. (C) Examples of cortical imaging frames in a Snap25-2A-GCaMP6s-D at 15-s intervals during CSD wave following 0 T sham application (C (i)) or 1.1 T sTMS application (C (ii)). Scale bar, 500 μm. (D) sTMS application reduced overall cortical fluorescence produced from the CSD wave, compared to sham stimulation. Time point 0 indicates an exponential increase in cortical fluorescence at the ROI, indicating the CSD wave. (E) Illustration of the properties of the CSD wave being further investigated. AUC = area under the curve. (F) sTMS application at 1.1 T significantly reduced the peak florescence of the CSD wave *versus* 0 T sham sTMS (*p* = 0.015). (G) sTMS application at 1.1 T had no significant change on the rise time from baseline to peak *versus* 0 T sham (*p* = 0.396). (H) sTMS application at 1.1 T had no significant difference on the velocity of the CSD wave *versus* 0 T sham (*p* = 0.289). (I) sTMS application at 1.1 T significantly reduced the AUC of the CSD wave (*p* = 0.007). (J) sTMS application at 1.1 T significantly reduced the decay of the CSD wave from peak back to baseline (*p* < 0.001)

In a separate set of experiments, we sought to assess the effect of sTMS on the CSD parameters (Fig. 2A, E), when eventually a CSD could be induced following active sTMS stimulation. Baseline GCaMP signal activity was recorded through the cortical window for 15 min before two 1.1-T sTMS pulses were applied to the visual cortex. Following at least a 10-min break, induction of a CSD was attempted every 10 min via mechanical stimulation of the cortex with a 30-G needle as previously performed. When a CSD was induced, cortical activity was recorded for 30 min post-CSD wave (Fig. 2A, B).

#### Spontaneous Cortical Neuronal Activity and sTMS

**Fig. 3:**
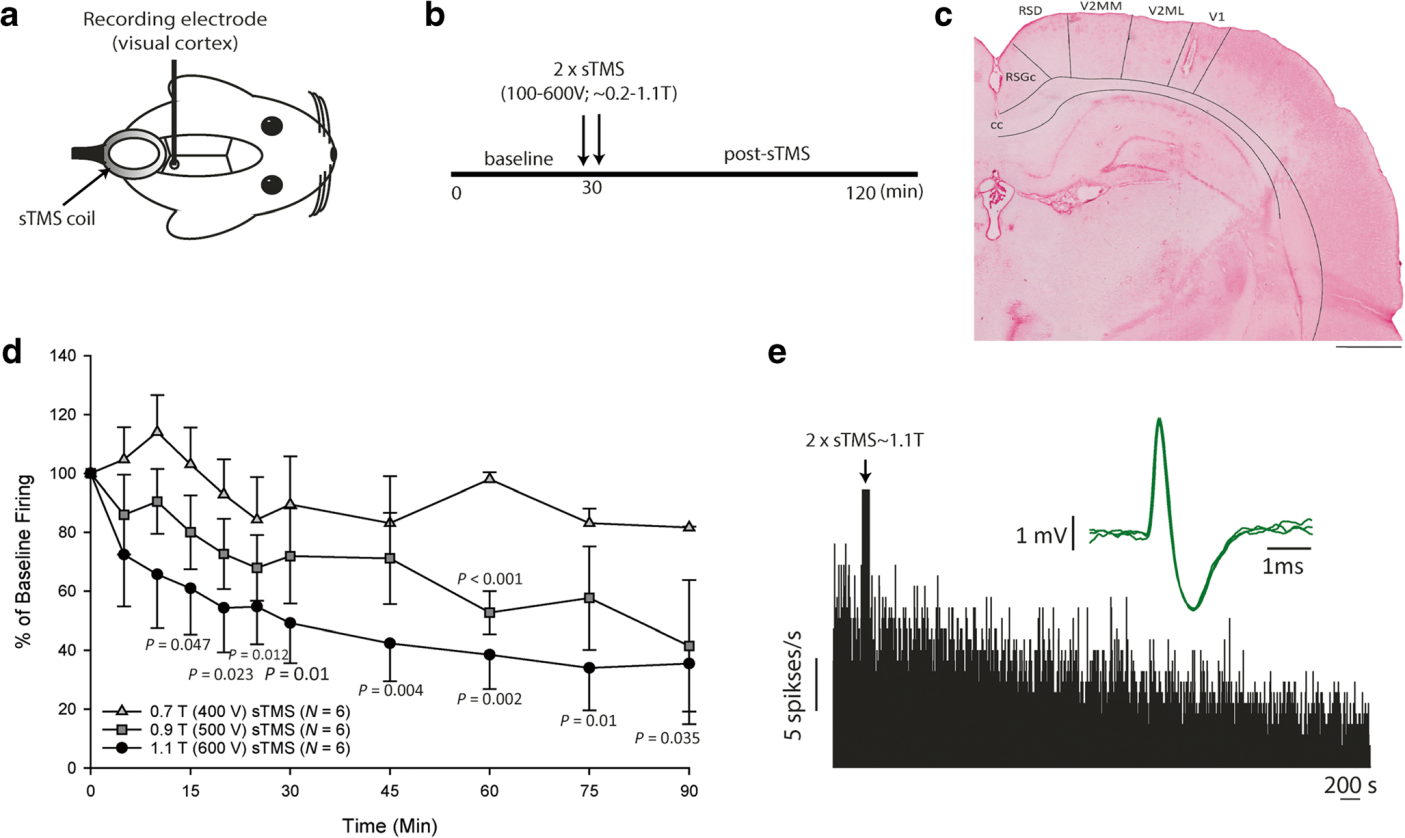
sTMS reduces spontaneous cortical neuronal firing in the visual cortex. (A) Experimental setup in the rat model: Extracellular single neuron activity was recorded from the visual cortex using a glass-insulated tungsten electrode. The sTMS coil was positioned above the visual cortex, close to the recording site. (B) A diagram illustrating the experimental protocol employed in the spontaneous cortical neuronal activity experiments. (C) Representative microphotograph of a lesion mark in the V1 visual cortex, demonstrating the recording location. (D) Two sTMS pulses at ~ 0.7 T (or at a lower intensity) had no significant effect on the spontaneous neuronal activity. At higher intensities (~ 0.9 and 1.1 T), sTMS significantly reduced the spontaneous neuronal activity recorded from neurons in the visual cortex. (E) Representative histogram of spontaneous neuronal firing demonstrating a reduction of activity post-sTMS (2 pulses at 1.1 T)

To assess the actions of sTMS on ongoing cortical activity, a stable baseline of spontaneous activity was recorded for at least 20 min from neurons in the visual cortex. Two sTMS pulses of intensity at 0.2–1.1 T were then randomly delivered, charging the stimulator at 100 V intervals up to 600 V (*N* = 6/group; total *N* = 36). Spontaneous activity was recorded for up to 90 min post-sTMS (Fig. 3A, B).

#### l-Glutamate-Evoked Excitability and sTMS

**Fig. 4:**
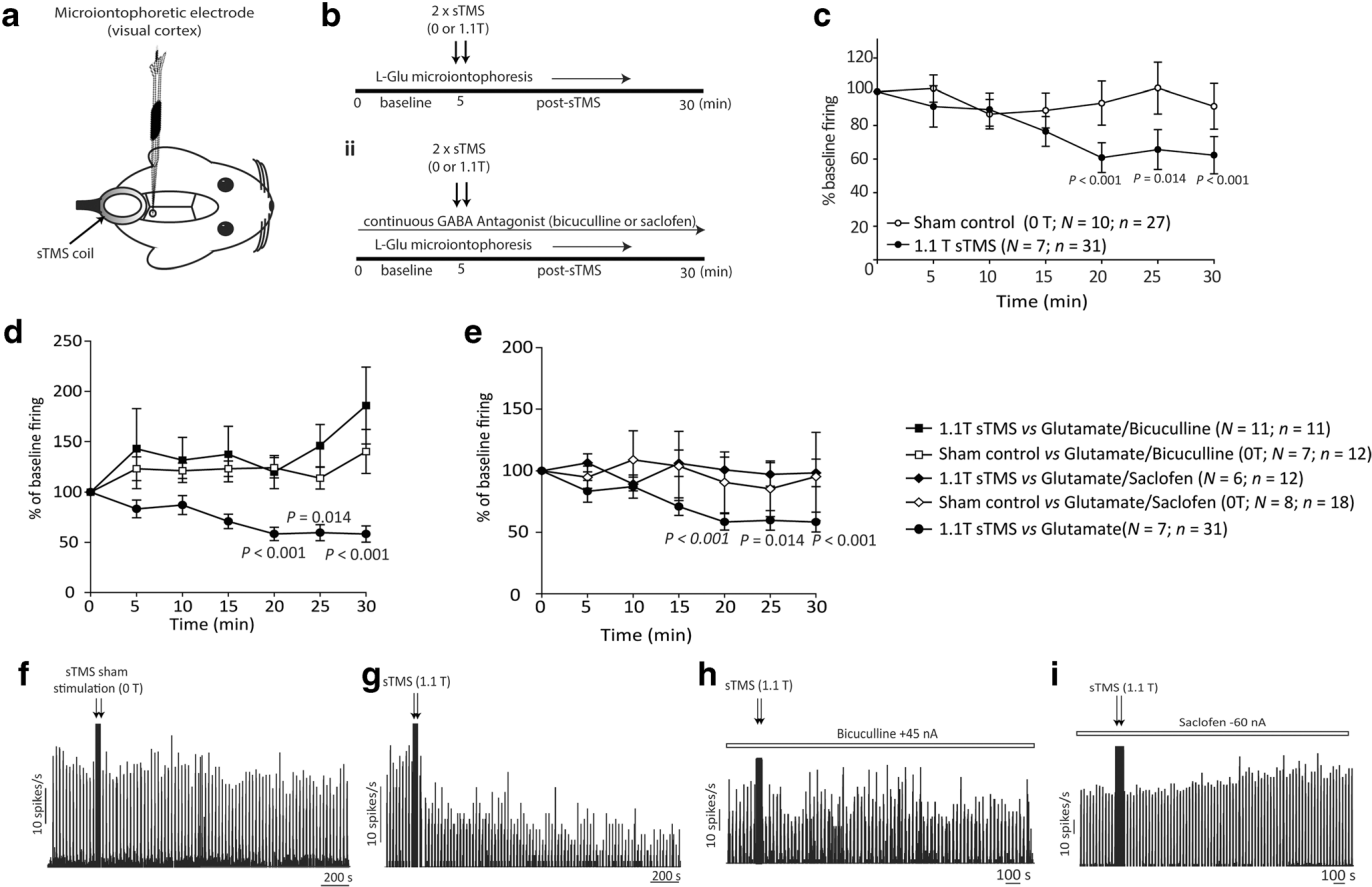
sTMS reduces glutamate-evoked neuronal firing, an effect blocked by GABA_A_ and GABA_B_ antagonists. (A) Experimental setup in the rat model: Cortical activity was recorded from single neurons into the visual cortex using a Carbostar recording/iontophoresis combination microelectrode. The sTMS coil was positioned above the occipital cortex, close to the recording site. (B) A diagram illustrating the experimental protocol employed in the l-glutamate-evoked cortical activity experiments i) in the absence of any GABA antagonist and ii) in the presence of constantly microiontophoresed GABA antagonists (bicuculline or saclofen). (C) sTMS at 1.1 T significantly reduced l-glutamate-evoked firing of cortical neurons, while sham stimulation at 0 T had no effect. (D) In the presence of the GABA_A_ antagonist bicuculline, sTMS at 1.1 T had no significant effect on the l-glutamate-evoked firing. Bicuculline alone did not change significantly l-glutamate-evoked firing. (E) In the presence of the GABA_B_ antagonist saclofen, sTMS at 1.1 T had no significant effect on the l-glutamate-evoked firing. Overall, saclofen alone did not change significantly l-glutamate-evoked firing. (F) Representative histogram of l-glutamate-evoked firing of a single cortical neuron. Sham sTMS stimulation (0 T) had no effect on l-glutamate-evoked firing. (G) Representative histogram of l-glutamate-evoked firing of single cortical neuron. Stimulation with 1.1-T sTMS pulses reduced evoked firing. (H) Representative histogram of l-glutamate-evoked firing of single cortical neuron in the presence of the GABA_A_ antagonist bicuculline. Stimulation with 1.1-T sTMS pulses had not effect on l-glutamate-evoked firing. (I) Representative histogram of l-glutamate-evoked firing of single cortical neuron in the presence of the GABA_B_ antagonist saclofen. Stimulation with 1.1-T sTMS pulses had no effect on l-glutamate-evoked firing

To assess the actions of sTMS on cortical glutamatergic excitability, microiontophoresis of l-glutamate was used to excite cortical neurons within the visual cortex. At least 5 min under baseline conditions were recorded. Two sTMS pulses (0-T sham stimulation or ~ 1.1-T active stimulation applied randomly) were then applied to the visual cortex. The train of l-glutamate pulses was continued for at least another 30 min post-sTMS application (Fig. 4A, B(i)).

To evaluate the influence of GABAergic activation as a potential mechanism of action of sTMS, in a separate set of experiments, either the GABA_A_ antagonist bicuculline or the GABA_B_ antagonist 2-hydroxysaclofen was constantly microiontophorised along with l-glutamate ejected in pulses. A stable baseline (at least 5 min) of l-glutamate-evoked activity was recorded in the presence of either bicuculline or saclofen and applied randomly, before two sTMS pulses (1.1 T or 0 T) were applied to the visual cortex. The train of l-glutamate pulses and the constant GABA antagonist ejections was continued for 30 min post-sTMS (Fig. 4B(ii)).

Upon completion of the experiment, PSB was ejected to mark the recording location. Brains were removed and stored in 4% paraformaldehyde until they were cut at 30-μm sections to identify the dye spot.

#### Electrically Induced CSD and sTMS

**Fig. 5:**
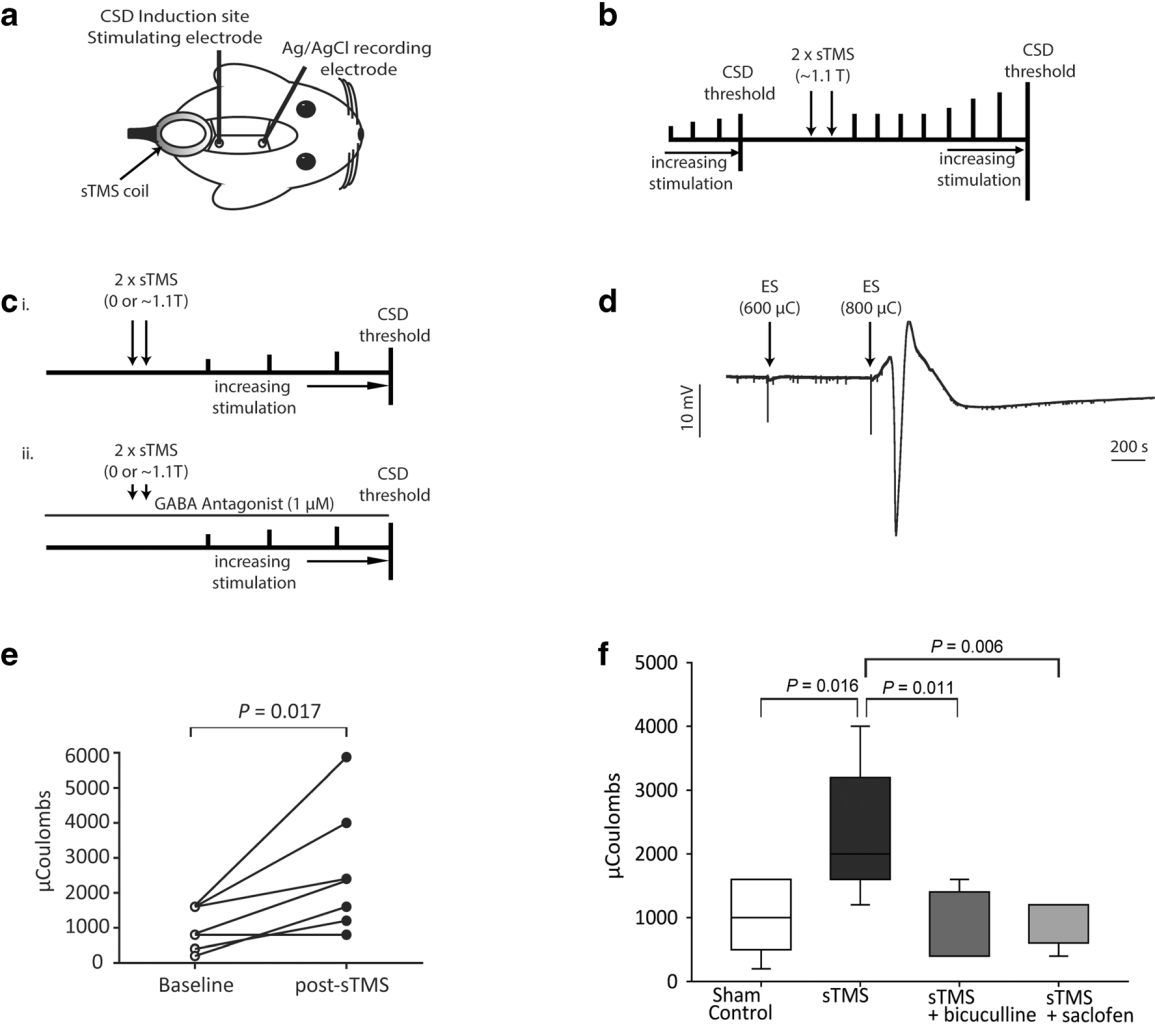
sTMS increases threshold for electrically induced cortical spreading depression (CSD), an effect blocked by GABA_A_ and GABA_B_ antagonists. (A) Experimental setup in the rat CSD model: Cortical steady potential (DC shift) was recorded in the visual cortex using a Ag/AgCl glass microelectrode. CSD was electrically induced at the visual cortex. The sTMS coil was positioned above the cortex, close to the CSD induction site. (B) Illustration of the experimental protocol employed in the electrically induced CSD model of migraine, in animals treated with sTMS following establishment of the CSD threshold at baseline, and repeated attempts for CSD induction post-sTMS. (C) Diagram illustrating i) the experimental protocol employed in the electrically induced CSD model of migraine, in animals pre-treated with sTMS at 0 T (sham stimulation) or at ~ 1.1 T, and ii) the experimental protocol employed in the electrically induced CSD model of migraine, in animals pre-treated with sTMS at 0 T (sham stimulation) or at ~ 1.1 T, in the presence of the GABA antagonists bicuculline and saclofen applied topically on the cortex. (D) Example of an electrically evoked CSD wave in an animal treated with sham sTMS. Electrical stimulation (ES) with 600 microcoulombs (μC) is sub-threshold; at 800 μC, a wave of CSD is evoked. (E) Two sTMS pulses at ~ 1.1 T significantly increased the electrical threshold required to generate a CSD, compared to the pre-sTMS baseline threshold (*p* < 0.05). (F) sTMS at ~ 1.1 T significantly increased the electrical threshold required to generate a CSD compared to the sham stimulation group (0 T) (*p* = 0.016), but not in the presence of the GABA_A_ and GABA_B_ antagonists bicuculline and saclofen

To identify if sTMS increases the threshold of activation for CSD induction, the migraine aura model of electrically induced CSD was utilised (Fig. 5A). The CSD threshold was first established in naïve animals under baseline conditions (*N* = 8). Sixty minutes later, two sTMS pulses (~ 1.1 T) were delivered over the visual cortex and induction of CSD using the same baseline stimulating parameters was attempted every 30 min up to 2 h post-sTMS stimulation. If CSD could not be induced using the pre-sTMS intensity, the threshold was re-examined by increasing stimulation current and duration until a CSD wave was induced (Fig. 5B).

In a separate group, animals were pre-treated with two sTMS pulses at 0 T (*N* = 8) or ~ 1.1 T (*N* = 8), applied randomly. Afterwards, amplitude and duration of the electrical stimulation were increased until a CSD wave was triggered [22] (Fig. 5C(i), D). Briefly, CSD threshold was found in microcoulombs (μC) by increasing duration (50 μs, 100 μs, 200 μs, 400 μs) and amplitude (0.5 mA, 1 mA, 2 mA, 3 mA, 4 mA, 5 mA) of electrical stimulus. The CSD induction threshold was found in animals pre-treated with two sTMS pulses (~ 0 T or 1.1 T; *N* = 8 per group) over the visual cortex before induction of a CSD and in animals that randomly received topical application of the GABA antagonist bicuculline (1 μM; *N* = 5) or 2-hydoxysaclofen (1 μM; *N* = 5) over the visual cortex prior to receiving two sTMS pulses (~ 1.1 T) (Fig. 5C(ii)).

### Data Analysis

#### *In Vivo* Imaging

Image (LIM images.nd2) files were exported from NIS-Elements (version 4.20) and analysed using ImageJ FIJI (version 1.52d). GCaMP fluorescence was assessed using five randomly selected 497.18 μm × 497.18 μm regions of interest (ROIs), avoiding blood vessels and unusually bright baseline fluorescence. Time lapse recordings were analysed using ImageJ FIJI. To normalise fluorescence intensity, Δ*F*/*F* was calculated as

$$ \Delta  F/F=\frac{F_t-{F}_0}{F_0} $$

where *F*_*t*_ is the fluorescence at time *t* and *F*_0_ is the fluorescence average over a baseline period [23]. Blood vessel diameters after the cortical events (sTMS and CSD) were normalised as a percentage of their baselines. Velocity of the CSD wave from the pinprick site to the ROI was recorded in addition to properties of the CSD wave: peak fluorescence, area under the curve, rise time and decay time (Fig. 2E). Effects of sTMS or CSD were analysed using a repeated measures analysis of variance (rmANOVA) for repeated measures followed by Student’s paired *t* test. Data were expressed as a mean of the baseline recordings ± SEM. Properties of the CSD waves were analysed using the Mann–Whitney *U* test.

#### Spontaneous Neuronal Activity Studies

For each unit, firing rate in spikes s^−1^ was averaged in 5-min intervals for 20 min pre-sTMS and up to 90 min post-sTMS. rmANOVA was computed with two factors: sTMS and repeats, to determine stimulation effects on spontaneous neuronal activity, followed by Student’s paired *t* test. When the assumption of sphericity with regard to the factor of repeats was violated, adjustments were made for the degrees of freedom and *p* values according to the Greenhouse–Geisser correction. All data were expressed as a mean percentage of baseline ± SEM, and significance was assessed at the *p* < 0.05 level (SPSS (version 23.0); SPSS Inc., USA).

#### l-Glutamate-Evoked Activity Studies

Statistical evaluations of the effect of sTMS on the neuronal response to microiontophoretic ejection of l-glutamate were made using the average rate of firing in Hz evoked during each epoch of microiontophoretic application. Five baseline pulses of l-glutamate were analysed to avoid variations of the responses of a cell between individual pulses, and the reliability of the measurements was tested using Cronbach’s alpha. The response of each cell under test conditions was examined as follows: a) l-glutamate (baseline) and b) l-glutamate post-sTMS or a) l-glutamate at the presence of bicuculline (baseline) and b) l-glutamate at the presence of bicuculline post-sTMS or a) l-glutamate at the presence of saclofen (baseline) and b) l-glutamate at the presence of saclofen post-sTMS. Effects of sTMS or sham stimulation (two sTMS pulses of 0 V) were analysed using an ANOVA for repeated measures followed by Student’s paired *t* test, using the average of the baselines of all tested parameters for comparisons, or were compared with vehicle control using an independent samples *t* test. When the assumption of sphericity with regard to the factor of repeats was violated, the Greenhouse–Geisser correction was applied. Data were expressed as a mean percentage of the baseline activity ± SEM.

#### Cortical Spreading Depression Studies

Within-group comparisons of electrical stimulation thresholds were analysed using the Wilcoxon signed-rank test. Effects of sTMS between groups were analysed using the Mann–Whitney *U* test. Significance was assessed at the *p* < 0.05 level. All data are expressed as the median value (μC) and interquartile range (IQ) (25–75% range).

## Results

### sTMS Had No Effect on Cortical GCaMP Fluorescence

To assess if the sTMS parameters used in the migraine treatment (~ 1.1 T) are actually exciting cortical neurons acutely, sTMS at 1.1 T (600 V) was applied over the visual cortex of Snap25-2A-GCaMP6s-D mice while GCaMP fluorescence was recorded. sTMS had no effect on GCaMP fluorescence (*p* = 0.134; Fig. 1C–E). As a positive control, needle prick–induced CSD at a later time point significantly changed GCaMP florescence (*F*_3.2, 156.8_ = 18.5, *p* < 0.05; Fig. 1C–E).

### sTMS Had No Effect on Cortical Blood Vessel Diameter

To assess the potential actions of sTMS on superficial blood vessels, the fluorescent dye dextran was injected in C57BL/6 mice. sTMS at 1.1 T was applied over the visual cortex of mice while dextran fluorescence was recorded. The diameter of blood vessels was measured pre- and post-sTMS. sTMS had no effect on blood vessel diameter (*p* ≥ 0.82; Fig. 1C, D, F). As a positive control, needle prick–induced CSD significantly altered the diameter of blood vessels (*F*_1.2, 6.1_ = 7.2, *p* < 0.05; Fig. 1C, F).

### sTMS Had a Significant Effect on Cortical Spreading Depression Characteristics

To assess the potential actions of sTMS on CSD once initiated following treatment with active sTMS stimulation, GCaMP fluorescence was recorded in Snap25-2A-GCaMP6s-D mice. sTMS significantly reduced the peak fluorescence (*N* = 6 per group, *p* = 0.015, *r* = − 0.31; Fig. 2D, F) and the area under the curve (*N* = 6 per group, *p* = 0.007, *r* = − 0.35; Fig. 2D, I) of the CSD wave compared to sham treatment. In addition, the resolution from peak back to baseline was also significantly reduced (*N* = 6 per group, *p* < 0.001, *r* = − 0.57; Fig. 2J). However, the rise time to peak was not significantly different (*N* = 6 per group, *p* = 0.40, *r* = − 0.11; Fig. 2G). The mean velocity of the CSD wave was not significantly different between 0 T sham and 1.1 T sTMS groups (*N* = 6 per group, *p* = 0.34, *r* = − 0.12; Fig. 2H).

### sTMS Inhibited Spontaneous Cortical Activity

A total of 36 cortical neurons were recorded in 36 animals. Only neurons that demonstrated stable spontaneous activity under baseline conditions were selected for treatment with two consecutive sTMS pulses. Overall cells were located at an average depth of 798 ± 53 μm below the cortical surface with an average frequency of spontaneous activity at a baseline of 7.2 ± 0.9 spikes s^−1^. sTMS applied at ~ 0.2 T (*N* = 6, *p* = 0.11), ~ 0.38 T (*N* = 6, *p* = 0.50), ~ 0.57 T (*N* = 6, *p* = 0.44) or 0.74 T (*N* = 6, *p* = 0.14; Fig. 3D) had no significant effect on spontaneous neuronal firing. sTMS applied at ~ 0.92 T significantly reduced spontaneous neuronal firing (*F*_2.7, 13.6_ = 3.5, *p* = 0.047), by a maximum of 48.3% at 60 min (*t*_5_ = 8.6, *p* < 0.001; Fig. 3D). The greatest reduction of spontaneous neuronal firing was seen after treatment with two pulses at ~ 1.1 T (*N* = 6, *F*_1.8, 7.4_ = 5.4, *p* = 0.037), with a maximum reduction of 66% occurring at 75 min post-sTMS (*t*_5_ = 4.59, *p* = 0.01; Fig. 3D, E). The maximum output used did not induce any motor responses.

### sTMS Inhibited l-Glutamate-Evoked Firing, but Not in the Presence of a GABA Antagonist

A total of 57 (in 25 animals) and 54 (in 20 animals) cortical neurons responding to l-glutamate microiontophoresis were recorded in the sham-treated (2 T × 0 T) and sTMS-treated (2 T × 1.1 T) groups, respectively. Neurons had an average frequency of spontaneous activity at the baseline of 10.3 ± 1.8 spikes s^−1^.

sTMS treatment of 2 pulses at 1.1 T was chosen based on outcomes from the dose–response studies on spontaneous neuronal activity presented above.

Between experimental groups, there was no difference across the mean firing of the five repeated epochs recorded during baseline (sham: *F*_4, 16_ = 0.98, *p* = 0.46; sTMS: *F*_2, 7_ = 0.73, *p* = 0.92; sTMS + bicuculline: *F*_4, 16_ = 1.22, *p* = 0.35; sTMS + saclofen: *F*_2, 26_ = 0.8, *p* = 0.48) and all baseline responses were reliable (Cronbach’s *α*, ≥ 0.88).

sTMS sham stimulation (2 T × 0 T, *n* = 27, *N* = 10 animals) had no effect on l-glutamate-evoked firing (*F*_2.9, 51.4_ = 1.4, *p* = 0.24; Fig. 4C, F). sTMS applied as two consecutive pulses at ~ 1.1 T significantly reduced l-glutamate-evoked firing, by 39.1% (*n* = 31, *N* = 7 animals, *F*_3.9, 100.9_ = 3.5, *p* = 0.011; Fig. 4C, G).

However, in the presence of the GABA antagonist bicuculline (*n* = 11, *p* = 7 animals) or saclofen (*n* = 12, *N* = 6), two pulses of sTMS at the same intensity had no effect on l-glutamate-evoked firing (bicuculline: *F*_2.1, 19.1_ = 0.77, *p* = 0.48; Fig. 4D, H; saclofen: *F*_2.4, 25.80_ = 0.80, *p* = 0.48; Fig. 4E, I). Sham stimulation (2 T × 0 T) in the presence of bicuculline (*n* = 12, *N* = 7 animals) or saclofen (*n* = 18, *N* = 8 animals) had no effect on l-glutamate-evoked firing (bicuculline: *F*_2.7, 30.0_ = 2.38, *p* = 2.38; saclofen: *F*_1.4, 9.8_ = 0.5, *p* = 0.6).

### sTMS Elevated Electrical Stimulation Threshold for Cortical Spreading Depression, but Not in the Presence of GABA Antagonists

The CSD threshold was determined at baseline conditions (median, 1200; IQ, 600–1600 μC). Following two consecutive sTMS pulses at ~ 1.1 T, induction of CSD was attempted every 30 min for 2 h until a CSD was induced (Fig. 5B). Only one animal produced CSD using the same cortical stimulating parameters 30 min after sTMS. In the remaining animals at 2 h post-sTMS, parameters for cortical stimulation were increased until a CSD wave was elicited (median, 2400; IQ, 1400–3200). Overall, sTMS treatment significantly increased by twofold the CSD induction threshold (*N* = 8, *p* = 0.017, *r* = − 0.84; Fig. 5E).

In a different experimental group, we determined the electrical stimulation threshold for CSD in the sham-treated group (*N* = 8; median, 1200; IQ, 500–1600 μC) and in the active sTMS-treated group (*N* = 8; median, 1600; IQ, 1600–3200 μC; Fig. 5C(i)). In the active sTMS treatment (2 T × 1.1 T), this threshold was significantly raised (sTMS *vs* sham: *p* = 0.016, *r* = − 0.85; Fig. 5F).

However, in the presence of GABA_A_ and GABA_B_ antagonists (Fig. 5C(ii)), two pulses of sTMS at the same intensity had no significant effect on the electrical stimulation threshold for CSD when compared to the sham group (sTMS + bicuculline *vs* sham: *N* = 5, *p* = 0.40, *r* = − 0.23; Fig. 5F; sTMS + saclofen *vs* sham: *N* = 5, *p* = 0.599, *r* = − 0.146). These CSD thresholds were significantly different compared to the electrical CSD threshold with active sTMS alone (sTMS + bicuculline *vs* sTMS: *N* = 13, *p* = 0.011, *r* = − 0.71; sTMS + saclofen *vs* sTMS: *N* = 13, *p* = 0.006, *r* = − 0.77; Fig. 5F).

## Discussion

The primary purpose of the study was to identify the cortical actions of the single-pulse transcranial magnetic stimulation (sTMS) treatment in migraine, a non-invasive neurostimulation technique. The data presented suggest that sTMS at these parameters does not excite cortical neurons nor influence cortical vascular tone. However, sTMS can reduce spontaneous neuronal activity in the visual cortex, potentially by engaging inhibitory GABAergic activity rather than directly supressing glutamatergic excitatory activity. These mechanisms also explain our findings that sTMS increased the threshold of induction of cortical spreading depression (CSD), a well-accepted animal model of migraine aura, given that sTMS had no effect in this model in the presence of GABA antagonists. The data do not exclude the possibility that alternative sTMS protocols would produce better results, but they do offer a significant advancement of our knowledge on the mechanism of action of sTMS in migraine treatment. In this study, we demonstrate an electrophysiological cortical mechanism of action of sTMS that most likely differs from the conventional sTMS mechanisms in other neurological fields and involves the GABAergic system. In addition, the effects of sTMS on cortical activity described here could expand to other conditions in which CSD plays at least some role, including epilepsy, transient ischemic attacks and traumatic brain injury.

Traditional TMS in a neurology clinic involved relatively large devices, with patients travelling to the hospital to receive treatment [24, 25]. Portable sTMS devices are essential for the more widespread use of this approach, and the migraine field is the first to have such a hand-held portable device available for its patients. sTMS has been shown to be effective in the acute treatment of migraine with and without aura [15, 16], as well as in the preventive treatment of migraine [17, 18]. Given that current treatment options are effective in only approximately 50% of migraine patients [26] and that cost barriers for some novel treatments may be prohibitive [27], non-invasive neuromodulation techniques may represent a good alternative treatment option for many migraine patients. Understanding the mechanism of action of sTMS in migraine is essential in understanding how a treatment acting solely in the CNS with minimal side effects can interfere with migraine pathobiology.

Migraine is considered a disorder of the brain with wide ramifications within the CNS [28]. The involvement of the occipital cortex in migraine pathophysiology has long been recognized. Migraine aura is a cortical phenomenon attributed to CSD initiated at the occipital cortex [29–32]. Furthermore, blood flow changes been recorded in migraine patients without aura, suggesting a functional role for the cortex [29]. Further, it is known that cortical hyperexcitability plays a defining role in migraine pathophysiology. Indeed, the induction of phosphenes, through occipital stimulation with high-intensity TMS (to increase cortical activity), showed a lower threshold in migraine patients compared to health controls [7, 33]. A study of photophobia during spontaneous migraine attacks using PET also found that this migraine symptom is linked with visual cortex hyperexcitability [34, 35]. It has been suggested that thalamocortical dysrhythmia in migraine patients may be responsible for abnormal cortical responses [36]. Of interest, an fMRI study of daily brain scans in a migraineur found a strong association for both hypothalamic and cortical activities during the premonitory phase of an attack [5]. Hence, a focal cortical treatment for migraine without systemic side effects, such as sTMS, is an attractive treatment opportunity. Andreou et al. [21] have previously shown that one pulse of sTMS with a rise time of 170 μs was more effective in blocking mechanically or chemically induced CSD compared to lower rise time pulses. They have also shown that with these parameters, sTMS can inhibit trigeminothalamic activity at the level of third-order thalamic neurons, but not in the trigeminocervical complex, suggesting a potential corticothalamic mechanism. However, the actual acute actions of sTMS on cortical activity have not previously been investigated. Here, we employed a much smaller coil of 11 mm diameter than what previously used in animal studies, which allowed a more focal stimulation [21, 37–39]. The smaller coil offered the advantage of positioning it nearer the recording site. We also used two consecutive pulses of sTMS as a treatment dose, given that clinically patients will also use at least two pulses as an acute treatment.

To address whether sTMS excites cortical neurons when used at the same rise time and intensity as in the human migraine device, we employed *in vivo* calcium imaging and applied sTMS over the cortex of GCaMP-expressing mice. GCaMP is a genetically encoded calcium indicator which increases its fluorescence intensity with the uptake of calcium, thereby providing a measure of neuronal activation. In these experiments, sTMS had no immediate or short-term effect on GCaMP fluorescent signal, suggesting that at these stimulating parameters, sTMS does not excite cortical neurons on average, at least in the superficial cortical layers. Potentially, the intensity of the magnetic stimulation used is not high enough to generate sufficient cortical activity. This is further evident by the fact that in none of the animals used in this study, sTMS application induced a CSD, which would have been an indication of increased cortical activity.

CSD itself is characterized as a slow wave of depolarisation of neurons and glial activation in the cortex, followed by a short-lasting depression. This phenomenon is accompanied by blood flow changes. Here, we employed *in vivo* confocal microscopy to visualize cortical blood vessels in dextran-injected mice, during and post-sTMS application. sTMS had no immediate or short-term effect on the vascular tone, suggesting that its efficacy in blocking CSD is purely by interactions with cortical cellular activity. While we were able to show a reduction in the CSD wave’s peak fluorescence and AUC following sTMS application, the root cause is unknown. Whether it is a consequence of a reduced population of cortical neurons having calcium influx from the CSD wave or a reduced calcium influx in all cortical cells remains unclear.

We found that sTMS can induce a dose-dependent inhibition of cortical spontaneous activity with the most effective stimulations being between 0.9 and 1.1 T, which is very close to the clinically used sTMS intensity. To explore whether this inhibitory effect was achieved by supressing excitatory cortical activity, we excited cortical neurons by microiontophoresis of glutamate and then applied the sTMS treatment. We found that indeed sTMS at ~ 1.1 T inhibits excitatory glutamatergic activity. However, it remained unclear if this occurs by direct influence on glutamatergic excitation or by engaging inhibitory connections that depress neuronal firing. To investigate this, the GABA_A_ and GABA_B_ receptor antagonists bicuculline and saclofen were continuously applied onto single neurons during pulsed excitation with l-glutamate. In these conditions, sTMS had no effect in blocking glutamatergic excitation, suggesting its effects are indirect by influencing the GABAergic system.

The sTMS-induced reduction of spontaneous and glutamatergic cortical activity found in our study may explain the sTMS actions on blocking CSD [21]. Our data suggest that this is achieved by increasing the threshold of CSD induction. The electrically evoked CSD model used in this study has the advantage of establishing the stimulation threshold needed to excite cortical neurons in order to induce a CSD wave. We show that indeed pre-treatment with sTMS increases the electrical threshold required to induce a CSD. We also show that the sTMS treatment can block for at least 2 h the induction of a CSD when using the baseline threshold electrical stimulation. However, in the presence of GABA_A_ and GABA_B_, sTMS has no effect in this CSD migraine model. These outcomes further confirm a mechanism of action for sTMS that involves GABAergic neurotransmission. Of interest, GABA agonists were previously shown to inhibit CSD in migraine models [40].

At the depth of around 800 μm we recorded from, we presume that recordings were made from large pyramidal neurons in lamina V, as interneurons, of various types, appear to be in more superficial laminae [41]. However, although every effort was made to record from neurons that displayed similar action potential characteristics, and as closed to a cell soma as possible (indicated by the biphasic shape of the action potential), the nature of such *in vivo* recordings does not allow us to be 100% certain of the type of neurons we recorded from. A major disadvantage of the electrophysiological experiments performed here was the long duration (typically several seconds) of the stimulus artefact produced by the sTMS device during capacitor discharge. This prevented us from confirming that indeed cortical neurons were not excited during the sTMS pulse. Given that sTMS was not found to excite cortical neurons in the GCaMP mice, its influence on GABAergic activity found in this study could potentially be through molecular changes at the receptor level at least, and not by activation of GABAergic neurons. Conversely, the *in vivo* GCaMP cortical imaging was able to image the superficial layers (I and II) of a large (~ 7.5 mm × 7.5 mm) cortical window in mice. Deeper layers were outside of the focus plane and, therefore, were only recorded as background noise. There is a possibility that neurons in deeper layers, particularly GABAergic neurons, are being activated but are out of focus. A study using voltage-sensitive dyes in cats found that sTMS at higher intensity applied at the visual cortex induced a brief focal activation, immediately followed by synchronous suppression of a large pool of neurons [42]. Using a different stimulating paradigm and a larger coil, Murphy et al. [43] suggested that sTMS activates inhibitory GABA fibres in the upper cortical layers which, in turn, inhibit the activity of dendritic pyramidal neurons in layer V. By blocking GABA_B_ receptors, they were able to prevent inhibitory effects of sTMS on the somatosensory cortex. Although in our *in vivo* experiments we could not record from dendrites or axons, activation of GABAergic fibres in the superficial layers remains a possibility and could explain the actions of sTMS seen in our study.

Due to the nature of the electrophysiological studies being performed here, blinding was not possible. However, the experimental protocols were designed to minimise the possibility of experimenter influence and randomisation of treatment intensity or GABAergic pharmacology was applied. The recording parameters and thresholds were set prior to the start of recording and were not altered during the experiment or analysis, thus allowing confidence in group comparisons. It should be also mentioned here that experiments were performed in anaesthetised animals for ethical reasons. Indeed, like most general anaesthetics, pentobarbital and urethane act on GABA_A_ receptors [44], which we found to be implicated in the sTMS mechanism of action. However, the use of anaesthetic did not prohibit the findings indicated here: In the local presence of a GABA antagonist, sTMS effects were blocked, suggesting that the use of anaesthesia, although it can significantly enhance the action of GABA_A_ receptors, would not significantly influence these outcomes. In addition, pentobarbital and urethane have comparable minimal effects on CSD frequency [45].

The results of this study demonstrate the acute cortical inhibitory actions of sTMS with parameters comparable to those that are effective in migraine treatment. The study further identifies the GABAergic system as an important biological mechanism in cortical inhibition and in determining susceptibility of CSD, the experimental correlate of migraine aura, to sTMS. Although the long-term action of sTMS in migraine prevention remains to be investigated, the current study provides important insights into the acute cortical modulation by sTMS in migraine. Cortical activity remains an important element of migraine pathophysiology, and sTMS offers a unique opportunity to modulate this in the absence of significant side effects that may be associated with other CNS treatments. The study of cortical modulation by sTMS will no doubt further illuminate the pathophysiology of migraine, the most disabling of the neurological disorder.

## Electronic Supplementary Material

ESM 1 (PDF 828 kb)

## Abbreviations

CSD: cortical spreading depression
sTMS: single-pulse transcranial magnetic stimulation
rTMS: repetitive transcranial magnetic stimulation

## References

[1] Vos T (2015). *Global, regional, and national incidence, prevalence, and years lived with disability for 301 acute and chronic diseases and injuries in 188 countries, 1990-2013: a systematic analysis for the Global Burden of Disease Study 2013*. Lancet.

[2] Murray CJ (2012). *Disability-adjusted life years (DALYs) for 291 diseases and injuries in 21 regions, 1990-2010: a systematic analysis for the Global Burden of Disease Study 2010*. Lancet.

[3] Headache Classification Committee of the International Headache Society, *The International Classification of Headache Disorders*, *3rd edition (beta version)*. Cephalalgia, 2013. **33**(9): p. 629-808. 10.1177/0333102413485658 23771276

[4] Goadsby PJ (2009). *Neurobiology of migraine*. Neuroscience.

[5] Schulte LH, May A (2016). *The migraine generator revisited: continuous scanning of the migraine cycle over 30 days and three spontaneous attacks*. Brain.

[6] Aurora SK (1998). *Transcranial magnetic stimulation confirms hyperexcitability of occipital cortex in migraine*. Neurology.

[7] Aurora SK (1999). *The occipital cortex is hyperexcitable in migraine: experimental evidence*. Headache.

[8] Hansen JM (2013). *Distinctive anatomical and physiological features of migraine aura revealed by 18 years of recording*. Brain.

[9] Bolay H (2002). *Intrinsic brain activity triggers trigeminal meningeal afferents in a migraine model*. Nature Medicine.

[10] Lambert GA, Truong L, Zagami AS (2011). *Effect of cortical spreading depression on basal and evoked traffic in the trigeminovascular sensory system*. Cephalalgia.

[11] Andreou AP, Sprenger T, Goadsby PJ (2012). *Cortical spreading depression-evoked discharges on trigeminothalamic neurons*. Headache.

[12] Lambru G, Giakoumakis E, Al-Kaisy A (2015). *Advanced technologies and novel neurostimulation targets in trigeminal autonomic cephalalgias*. Neurol Sci.

[13] Miller S, Matharu M (2017). *Non-invasive Neuromodulation in Primary Headaches*. Curr Pain Headache Rep.

[14] Barker AT, Jalinous R, Freeston IL (1985). *Non-invasive magnetic stimulation of human motor cortex*. The Lancet.

[15] Lipton RB (2010). *Single-pulse transcranial magnetic stimulation for acute treatment of migraine with aura: a randomised, double-blind, parallel-group, sham-controlled trial*. Lancet Neurol.

[16] Bhola R (2015). *Single-pulse transcranial magnetic stimulation (sTMS) for the acute treatment of migraine: evaluation of outcome data for the UK post market pilot program*. J Headache Pain.

[17] Starling AJ (2018). *A multicenter, prospective, single arm, open label, observational study of sTMS for migraine prevention (ESPOUSE Study)*. Cephalalgia.

[18] Lambru G (2018). *SINGLE-PULSE TRANSCRANIAL MAGNETIC STIMULATION (STMS) FOR THE TREATMENT OF MIGRAINE: A PROSPECTIVE REAL WORLD EXPERIENCE*. Cephalalgia.

[19] Liston C (2014). *Default mode network mechanisms of transcranial magnetic stimulation in depression*. Biological Psychiatry.

[20] Singh A (2019). *Personalized repetitive transcranial magnetic stimulation temporarily alters default mode network in healthy subjects*. Scientific Reports.

[21] Andreou AP (2016). *Transcranial magnetic stimulation and potential cortical and trigeminothalamic mechanisms in migraine*. Brain.

[22] Ayata C (2006). *Suppression of cortical spreading depression in migraine prophylaxis*. Ann Neurol.

[23] Chisholm, K.I., et al., *Large scale in vivo recording of sensory neuron activity with GCaMP6*. eNeuro, 2018. **5**(1). 10.1523/ENEURO.0417-17.2018 PMC5898788 29662940

[24] Barker AT, Jalinous R, Freeston IL (1985). *Non-invasive magnetic stimulation of human motor cortex*. Lancet.

[25] Brighina F (2004). *rTMS of the prefrontal cortex in the treatment of chronic migraine: a pilot study*. J Neurol Sci.

[26] Andreou AP, Goadsby PJ (2010). *Topiramate acts on kainate receptors within the trigeminothalamic pathway*. Headache.

[27] Reinke T (2018). *Aimovig for Migraine Prevention: The New Kid May Have Trouble Fitting in*. Manag Care..

[28] Goadsby P (2009). *Neurobiology of migraine*. Neuroscience.

[29] Olesen J (1998). *Regional cerebral blood flow and oxygen metabolism during migraine with and without aura*. Cephalalgia.

[30] Olesen J (1990). *Timing and topography of cerebral blood flow, aura, and headache during migraine attacks*. Ann Neurol.

[31] Arngrim N (2017). *Heterogenous migraine aura symptoms correlate with visual cortex functional magnetic resonance imaging responses*. Ann Neurol.

[32] Hadjikhani N (2001). *Mechanisms of migraine aura revealed by functional MRI in human visual cortex*. Proc Natl Acad Sci U S A.

[33] Mulleners WM (2001). *Visual cortex excitability in migraine with and without aura*. Headache.

[34] Denuelle M (2011). *A PET study of photophobia during spontaneous migraine attacks*. Neurology.

[35] Brennan KC (2011). *Turn down the lights!: an irritable occipital cortex in migraine without aura*. Neurology.

[36] Coppola G, Pierelli F, Schoenen J (2007). *Is the cerebral cortex hyperexcitable or hyperresponsive in migraine?*. Cephalalgia.

[37] Ambriz-Tututi M, Sanchez-Gonzalez V, Drucker-Colin R (2012). *Transcranial magnetic stimulation reduces nociceptive threshold in rats*. J Neurosci Res.

[38] Ji RR (1998). *Repetitive transcranial magnetic stimulation activates specific regions in rat brain*. Proc Natl Acad Sci U S A.

[39] Yang Y (2007). *Sex differences in antidepressant-like effect of chronic repetitive transcranial magnetic stimulation in rats*. Prog Neuropsychopharmacol Biol Psychiatry.

[40] Holland PR, Akerman S, Goadsby PJ (2010). *Cortical spreading depression-associated cerebral blood flow changes induced by mechanical stimulation are modulated by AMPA and GABA receptors*. Cephalalgia.

[41] Fröhlich, F., *Microcircuits of the Neocortex*, in *Network Neuroscience*, F. Fröhlich, Editor. 2016, Academic Press- Elsevier Inc.: North Carolina, US. p. 85-95.

[42] Kozyrev V, Eysel UT, Jancke D (2014). *Voltage-sensitive dye imaging of transcranial magnetic stimulation-induced intracortical dynamics*. Proc Natl Acad Sci U S A.

[43] Murphy SC (2016). *Transcranial magnetic stimulation (TMS) inhibits cortical dendrites*. Elife.

[44] Garcia PS, Kolesky SE, Jenkins A (2010). *General anesthetic actions on GABA(A) receptors*. Curr Neuropharmacol.

[45] Kudo C (2008). *The impact of anesthetics and hyperoxia on cortical spreading depression*. Experimental Neurology.

